# Case Report: Fulminant Myocarditis Successfully Treated With Extracorporeal Membrane Oxygenation in Ikeda Strain *Orientia tsutsugamushi* Infection

**DOI:** 10.3389/fcvm.2021.795249

**Published:** 2021-12-22

**Authors:** Hyejin Park, Yongwhan Lim, Min Chul Kim, Seong Eun Kim, In-Seok Jeong, Yoo Duk Choi, Dong-Min Kim

**Affiliations:** ^1^Department of Internal Medicine, Chonnam National University Medical School, Chonnam National University Medical Hospital, Gwangju, South Korea; ^2^Department of Cardiovascular Medicine, Chonnam National University Medical School, Chonnam National University Medical Hospital, Gwangju, South Korea; ^3^Division of Infectious Disease, Department of Internal Medicine, Chonnam National University Medical School, Chonnam National University Medical Hospital, Gwangju, South Korea; ^4^Department of Cardiothoracic Surgery, Chonnam National University Medical School, Chonnam National University Medical Hospital, Gwangju, South Korea; ^5^Department of Pathology, Chonnam National University Medical School, Gwangju, South Korea; ^6^Department of Internal Medicine, College of Medicine, Chosun University, Gwangju, South Korea

**Keywords:** fulminant myocarditis, scrub typhus, *Orientia tsutsugamushi*, extracorporeal membrane oxygenation, case report

## Abstract

Scrub typhus is an acute zoonotic febrile illness caused by *Orientia tsutsugamushi* having a specific geographic endemic area. This infection could be complicated with multi-organ involvement including myocarditis with variable severity. Here, we report a rare case of scrub typhus with biopsy-proven acute fulminant myocarditis which progressed very rapidly to cardiac arrest and was treated successfully with extracorporeal cardiopulmonary resuscitation. Clinicians should be alert to possible rapid progression of scrub typhus myocarditis to fulminant form and be prepared for close monitoring and temporary mechanical support if indicated.

## Introduction

Scrub typhus is an acute zoonotic febrile illness caused by *Orientia tsutsugamushi*, an obligatory intracellular bacterium transmitted to humans through bites of a chigger mite belonging to *Leptotrombidium* species (1). Although it has been thought endemic disease in many countries in the Asia-Pacifica area called the tsutsugamushi triangle which includes Korea, Japan, China, Taiwan, India, Indonesia, Thailand, Sri Lanka, and the Philippines (2), there have been some case reports of scrub typhus from outside of the traditional endemic area (3, 4). Scrub typhus could be complicated with multi-organ involvement including pneumonia, acute kidney injury, meningoencephalitis, gastrointestinal bleeding, and myocarditis (5, 6) especially in delayed proper antibiotic administration (7). Among them, myocarditis is an uncommon cardiac manifestation with variable severity from a mild form to fulminant myocarditis which has been reported only in a few cases (8).

In this article, we presented a very rare case of biopsy-proven acute fulminant myocarditis manifested as a cardiac arrest because of very rapid progression and treated successfully with venoatrial (VA) extracorporeal membrane oxygenation (ECMO) in a case of scrub typhus by Ikeda strain of *O. tsutsugamushi*. Clinicians should be alert to the possibility of rapid progression to fulminant myocarditis in patients presenting suggestive signs of myocarditis in scrub typhus.

## Case Description

**Figure 1:**
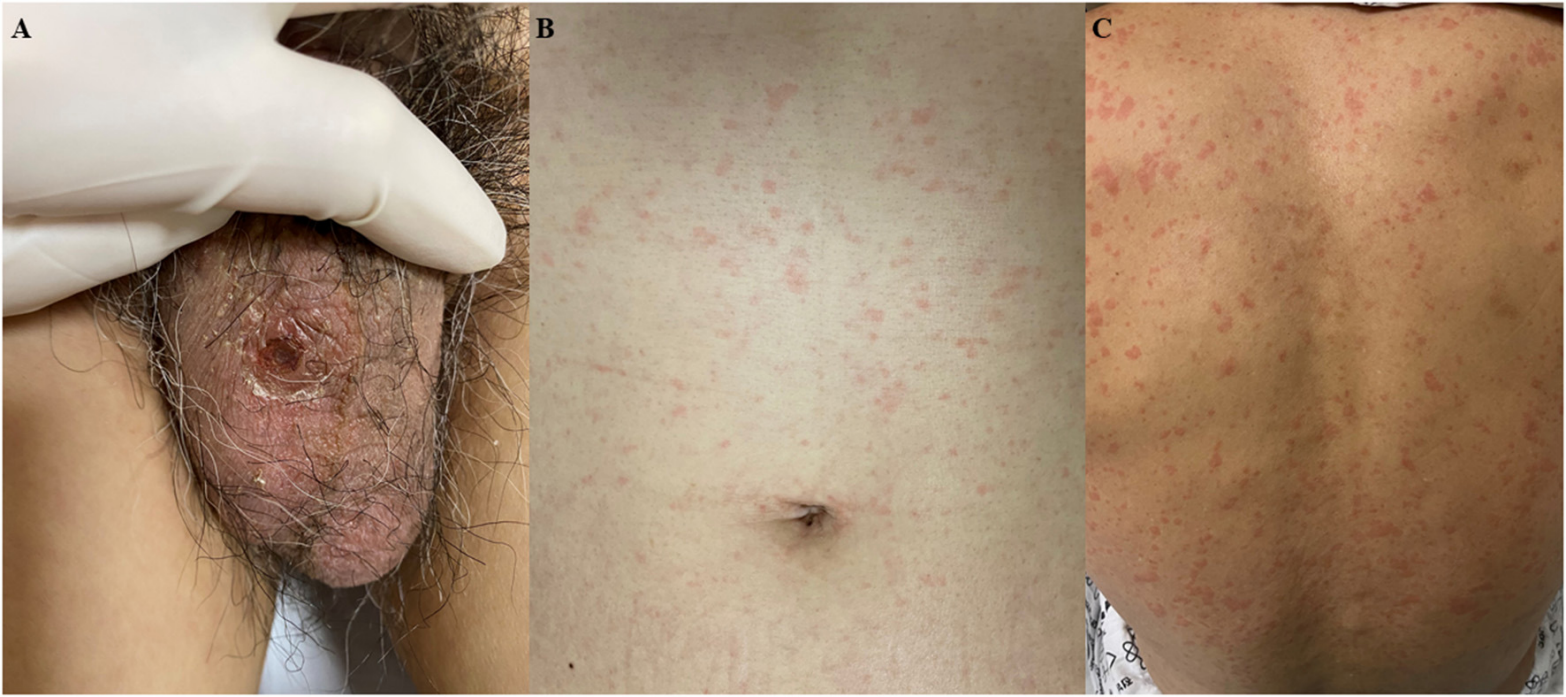
Skin lesions at presentation. **(A)** 1 × 1 cm sized erythematous to black colored ulcerative lesion covered with crust was observed on his scrotum. **(B,C)** Diffuse maculopapular rash on the trunk and back.

A 61-year-old previously healthy male patient was transferred from a private hospital because of recurrent fever which started 10 days ago during the summer period in South Korea. He also complained of orthopnea and oliguria starting 4 days ago. On the initial physical examination at the emergency department, a fine inspiratory crackle was heard on both lungs, and an asymptomatic 1 × 1 cm sized erythematous to black-colored ulcerative lesion covered with crust was observed on his scrotum (Figure 1A). There was also a diffuse maculopapular rash on his trunk (Figure 1B) and back (Figure 1C). We found he had a history of sitting on the lawn for several hours about 20 days ago. Chest x-ray showed multifocal haziness on both lungs, and initial laboratory test showed upper normal range of white blood cells (9,800/ul) with neutrophil dominance (93.1%), elevated CRP (28.59 mg/dl), procalcitonin (30 ng/ml), and his serum creatinine was also elevated up to 2.84 mg/dL compared to 1.1 mg/dl of creatinine at his baseline obtained 4 days ago. Because his ECG showed slight ST-segment depression on the V5-V6 (Supplementary Figure 1A) and troponin T was elevated up to 0.231 ng/ml (reference value < 0.005 ng/ml), echocardiography was performed, and it showed focal hypokinesis in basal, basoinferior, and mid to basal inferolateral segments with mild left ventricular (LV) systolic dysfunction with 45% of ejection fraction (EF) with normal LV wall thickness (**Figure 3A**, Supplementary Videos 1, 2). Clinical findings of the patient suggested multi-organ involvement including pneumonia, acute kidney injury (AKI), and possible myocarditis. Other laboratory test results are summarized in the Supplementary Table 1.

**Figure 2:**
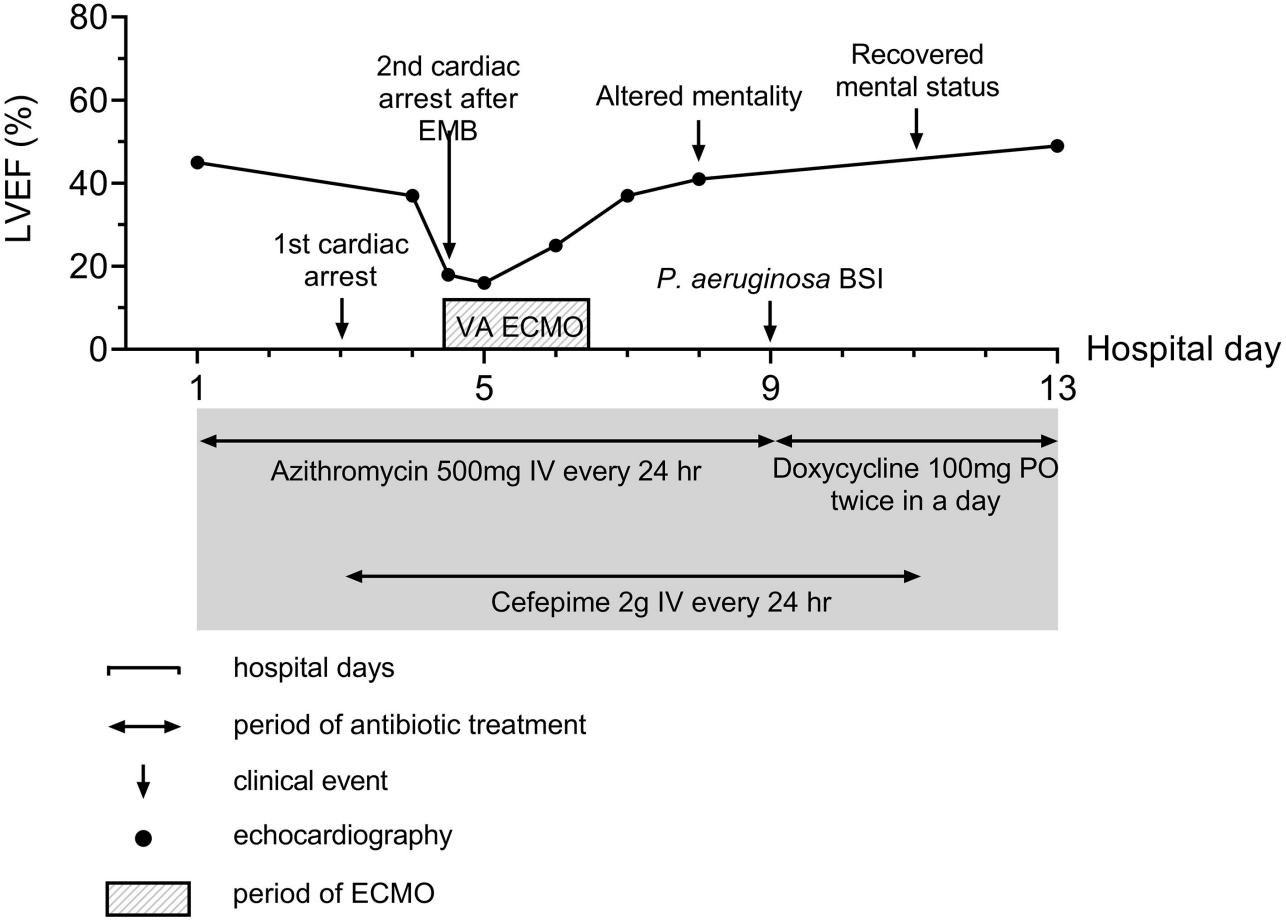
Summary of disease course. Major events and therapeutics are summarized in the figure. BSI, Bloodstream infection; ECMO, Extracorporeal membrane oxygenation; EMB, endomyocardial biopsy.

Although summer is not the peak season of scrub typhus in Korea and the result of the initial antibody for *O. tsutsugamushi* was negative, the clinical diagnosis of scrub typhus was made based on eschar, typical skin rash (Figures 1A–C), and history of outdoor activity. Administration of intravenous (IV) azithromycin was started (500 mg every 24 h) and information about major events and treatments during the clinical course is summarized in Figure 2.

**Figure 3:**
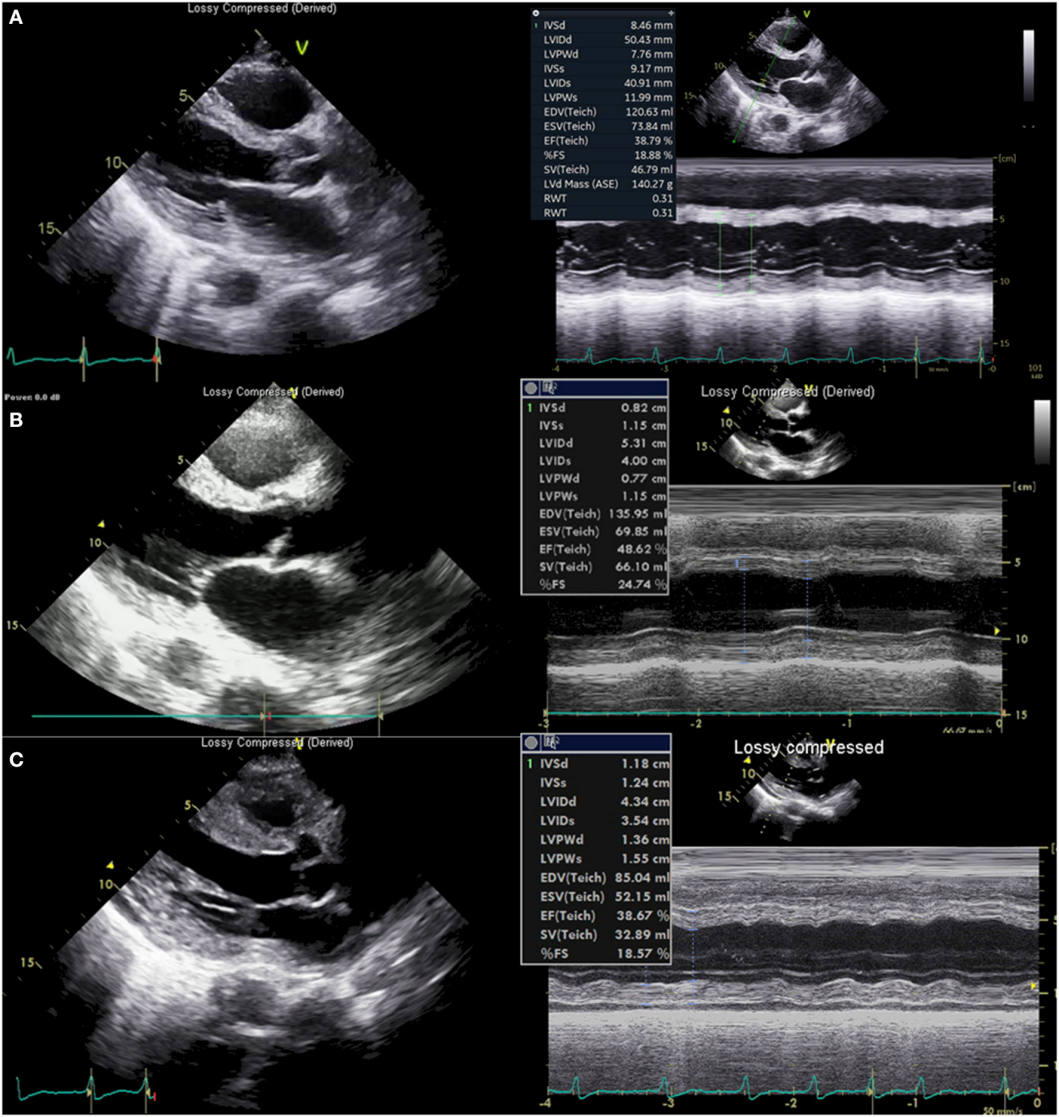
Echocardiography. **(A)** Echocardiography at presentation showed normal left ventricular (LV) wall thickness and chamber size. **(B)** Echocardiography 6 h before cardiac arrest showed normal LV wall thickness. **(C)** Echocardiography just after extracorporeal CPR showed thickened LV (13.6 mm, posterior wall, 11.8 mm septum) and right ventricle wall.

On the third day of hospitalization, pneumonic infiltration was aggravated on chest x-ray and intravenous cefepime (2 g every 24 h) was initiated to treat hospital-acquired pneumonia and used from hospital day 3 to 11. Oxygen demand rapidly increased and renal function has deteriorated. Subsequent cardiac arrest occurred. The patient was resuscitated with 25 min of conventional cardiopulmonary resuscitation (CPR). Because the cardiac arrest was thought of as respiratory origin related to pneumonia and AKI, mechanical ventilation and continuous renal replacement therapy were started after the patient was transferred to the intensive care unit (ICU). On the next day (hospital day four), troponin I was markedly elevated from 2.771 ng/ml 1 day ago up to 9.383 ng/ml, and there was newly developed T wave inversion on V1-V3 (Supplementary Figure 1B). Follow-up echocardiography showed global hypokinesis with aggravated LV systolic function (EF = 37%) with normal LV and RV wall thickness (Figure 3B, Supplementary Videos 3, 4). Because the progression of myocarditis was suspected, endomyocardial biopsy (EMB) from the RV and coronary angiography (CAG) was performed 4 h later. Coronary angiography did not show any significant lesion.

Just before moving the patient to an ICU from a cath lab, his heart rate became slower to about 40/min with junctional rhythm, and blood pressure was lowered to 80–90 mmHg of systolic blood pressure. Cardiac arrest suddenly occurred again. Despite 10 min of conventional CPR, return of spontaneous circulation was not achieved, and rhythm during CPR was pulseless electrical activity (PEA), thus non-shockable. Extracorporeal CPR (ECPR) was started, and cannulation was successfully performed with a 15 French perfusion cannula in the right common femoral artery and a 21 French drainage cannula in the common femoral vein of the same side. VA-ECMO pump on was done after 24 min of total low flow time. Echocardiography performed just after ECMO pump on showed markedly thickened up to about 13 mm (Figure 3C, Supplementary Video 5) both ventricular wall with newly formed mild to moderate pericardial effusion without features of cardiac tamponade. Left ventricular function was compromised severely with 14% of EF. Such changes had developed just several hours after the echocardiography on the same day.

Although echocardiography on the next day showed slightly better cardiac function with 18% of LVEF without changes of the amount of pericardial effusion pulmonary edema was refractory to medical measures. Left atrial cannulation *via* septostomy using a 21 French drainage cannula via the left common femoral vein was performed for the purpose of left heart unloading, and it ameliorated pulmonary edema. He recovered alert mental status without any neurologic deficit on the same day.

**Figure 4:**
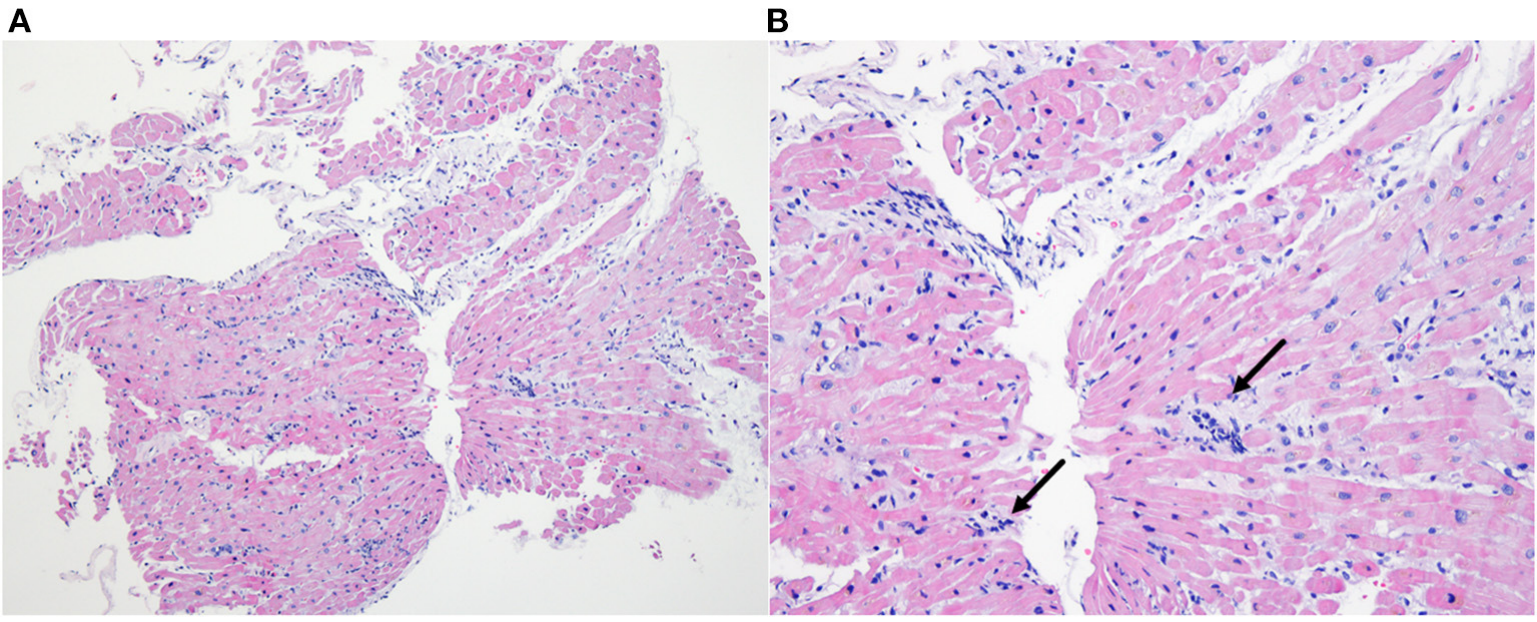
Endomyocardial biopsy. Inflammatory cell infiltration consisting of T-lymphocyte within interstitium and myocardium (**A**-X40, Hematoxylin-Eosin) (**B**-X200, Hematoxylin-Eosin), with an indication of lymphocyte (black arrow).

During VA-ECMO treatment, we obtained the positive result of PCR for *O. tsutsugamushi*. The whole blood sample was referred to the Infection Research Laboratory of Chosun College of Medicine on hospital day 3. Whole blood (WB) sample was used for the nested PCR test. DNA was prepared from the WB (0.3 ml) of the patient using the DNeasy Blood & Tissue kit (Qiagen, Hilden, Germany). A size of 483 bp product was obtained by nested PCR on a part of the 56 kDa gene of *O. tsutsugamushi*. Ikeda strain of *O. tsutsugamushi* was detected by DNA sequencing (GenBank accession number AP008981). Endomyocardial biopsy showed lymphocytic infiltration in myocardium and interstitium which is compatible with myocarditis with T-lymphocytic infiltration (Figures 4A,B) with positive staining for CD3 and CD5 and negative for CD 20. Other tests for viral or autoimmune etiologies were negative. Acute fulminant myocarditis caused by scrub typhus was confirmed.

Cardiac function was recovered up to 38% of EF on the third day of ECMO treatment, weaning from ECMO and the mechanical ventilator was possible for 3 and 4 days, respectively, after starting ECMO treatment. The patient was not treated with an immune-modulating agent including glucocorticoid or intravenous immunoglobulin during the period.

His mental status, however, was changed. Although the patient seemed alert, he showed global aphasia. Brain magnetic resonance imaging did not show any abnormalities. The cerebrospinal fluid exam showed mild lymphocytic pleocytosis (WBC 65/μl, 92.3% of lymphocyte), slightly increased protein (78.7 mg/dl), and normal glucose (81 mg/dl). CSF Adenosine deaminase was 20.3 IU/L. CSF culture and PCR results using CSF were negative for any microorganisms. It was suggesting meningoencephalitis complicated by scrub typhus. Doxycycline (100 mg twice a day) was started because of the unclear CSF concentration of intravenous azithromycin. Three days later, he got his own mental status, and global aphasia was totally resolved.

The patient was transferred to a general ward and discharged after about 10 days without other clinical events. Antibody for *O. tsutsugamushi* was converted positive in the hospital day 19 by immunofluorescence assay. The *O. tsutsugamushi* Ig G titer was 1:2,048 and Ig M was 1:128. Follow-up echocardiography just before discharge showed more improved left ventricular function (EF = 54%) with normal LV wall thickness (Supplementary Video 6) and he was discharged with a prescription of cardioprotective agents including bisoprolol, valsartan, spironolactone, and furosemide.

## Discussion

This case is scrub typhus with multi-organ involvement including biopsy-proven acute fulminant myocarditis which progressed very rapidly despite appropriate antibiotic treatment. The clinical message that we could get from this case is very clear-myocarditis complicating scrub typhus could progress to fulminant myocarditis very rapidly and measures for hemodynamic support including mechanical circulatory support on time is central to save patients.

In this case, we could suppose the reasons for fulminant myocarditis in two aspects. The patient visited a private clinic before being transferred to our institution, but clinical diagnosis of scrub typhus was not considered so a proper antibiotic was initiated 10 days from symptom onset. The incidence of complicated scrub typhus increases when treatment of scrub typhus is delayed in previous studies (7). Delayed diagnosis is thought to be related to the uncommon seasonal occurrence in this patient. In Korea, the peak season of scrub typhus is known to be September to December and 90% of reported cases to occur during this period (Korea Centers for Disease Control and Prevention) (19).

Another reason for complicated scrub typhus is related to the Ikeda strain of *O. tsutsugamushi*. Ikeda strain belongs to the Japanese Gilliam serotype and is known to be highly virulent in animal experiments (9). Chang et al. (10) reported 79% were typed as Boryong and 15% as Karp from the study *of O. tsutsugamushi* isolates from humans in Korea. The unusual fulminant myocarditis could be explained by hypervirulent Ikeda strain in another aspect.

The incidence and clinical impact of myocarditis in scrub typhus have been variably reported. It was reported from 2.4 to 14% (5, 11, 12), and one systematic review showed 4.3% of incidence (11). The mortality of scrub typhus myocarditis in ICU patients was reported 15.5% (12). In a systematic review that dealt with a prognosis of untreated scrub typhus, the reported difference of mortality according to the presence of complicated myocarditis was 20% (11). This variable incidence and impact on mortality might reflect the heterogeneity of the study population and diagnostic approach in those studies. For example, Kim et al. reported 2.4% of the incidence of myocarditis in scrub typhus in their cohort which was recruited regardless of disease severity (5). In study enrolled only severe, complicated cases of scrub typhus who were admitted to ICU and dedicated to myocarditis (12) reported 14% of the incidence of myocarditis with association with higher mortality.

These differences in reported incidence and impact on the clinical course of myocarditis in scrub typhus also might reflect variable severity of myocarditis itself. The severity or course of myocarditis seems to be ranged from mild one (13, 14) to fulminant one like ours and cases reported by only one study (8). In addition, the nature of scrub typhus itself with possible multi-organ involvement in complicated cases might mask the fulminant nature of myocarditis in scrub typhus.

The mechanism of myocarditis in scrub typhus is not well established. In the present case, we tried *O. tsutsugamushi* PCR and *O. tsutsugamushi* antibody immunohistochemical stain using EMB tissue to reveal the mechanism of myocarditis was an invasion of the pathogen itself or immunologic reaction but failed. Further study is needed to elucidate the mechanisms of myocarditis in scrub typhus.

In our case, which is the first biopsy-proven acute fulminant myocarditis in scrub typhus, could provide a clear profile of fulminant myocarditis in this infection. To our best knowledge, there have been only two biopsies, not an autopsy, proven myocarditis in scrub typhus, which was reported in 1991 by the Japanese group (13) and in 1996 by the Korean group (15). Although these previously reported cases were classified as lymphocyte myocarditis based on biopsy findings like our case, these were not fulminant cases and responded well to antibiotics treatment without severe hemodynamic compromise. On the contrary, the patient in our case showed very rapid progression within several hours to fulminant myocarditis and cardiac arrest despite appropriate antibiotics usage. Such progression was assessed with multiple times echocardiography which showed dramatic changes in systolic function and wall thickness in both ventricles. In addition, the patient was also complicated multi-organ involvement including AKI, pneumonia, and meningoencephalitis before and after the progression to fulminant myocarditis, and such a complicated clinical situation might make the diagnostic approach for fulminant myocarditis difficult on time. At last, in the case of fulminant progression which does not respond to medical management, successful and rapid implantation of VA ECMO on time was critical to saving the patient. Experience of ECMO in fulminant myocarditis caused by scrub typhus seems limited and only a few reports are available (16). Clinicians, however, should be alert to the possibility of rapid progression of scrub typhus myocarditis and be prepared for temporary mechanical support (MCS) based on the evidence provided by some studies (17, 18).

## Conclusion

We reported a very rare case of biopsy-proven-acute fulminant myocarditis in scrub typhus manifested as cardiac arrest and treated successfully with VA ECMO. Clinicians should keep in mind the possibility of rapid progression of myocarditis in scrub typhus, even during several hours, and should perform essential evaluation and close monitoring to provide appropriate treatment including temporary MCS if indicated to save patients.

## Data Availability Statement

The datasets presented in this study can be found in online repositories. The names of the repository/repositories and accession number(s) can be found in the article/Supplementary Material.

## Ethics Statement

Ethical review and approval was not required for the study on human participants in accordance with the local legislation and institutional requirements. The patients/participants provided their written informed consent to participate in this study. Written informed consent was obtained from the individual(s) for the publication of any potentially identifiable images or data included in this article.

## Author Contributions

HP, YL, SK, and MK conceived inspiration together. HP gathered patient information. HP and YL equally contributed to the initial draft of this manuscript. HP, YL, and MK edited the initial manuscript together in the cardiologic aspect. I-SJ added and edited the part of the manuscript related to ECPR and ECMO. SK wrote the whole part of the manuscript related to infectious disease. D-MK edited the part of the manuscript related to the diagnostic method of scrub typhus. YC selected the pathologic image and wrote the caption for it. MK and SK orchestrated the whole process for writing the manuscript and completed the final version of this draft. All authors contributed to the article and approved the submitted version.

## Conflict of Interest

The authors declare that the research was conducted in the absence of any commercial or financial relationships that could be construed as a potential conflict of interest.

## Publisher's Note

All claims expressed in this article are solely those of the authors and do not necessarily represent those of their affiliated organizations, or those of the publisher, the editors and the reviewers. Any product that may be evaluated in this article, or claim that may be made by its manufacturer, is not guaranteed or endorsed by the publisher.

## Supplementary Material

Supplementary Figure 1 **(A)** EKG at presentation showed slight ST-segment depression on the V5-V6. **(B)** EKG on hospital day 3 showed newly developed T wave inversion on V1-V3.

Supplementary Table 1 Laboratory findings at initial presentation.

Supplementary Video 1 Portable echocardiography at presentation, apical four-chamber view.

Supplementary Video 2 Portable echocardiography at presentation, apical two-chamber view.

Supplementary Video 3 Portable echocardiography on day 4, apical four-chamber view.

Supplementary Video 4 Portable echocardiography on day 4, apical two-chamber view.

Supplementary Video 5 Portable echocardiography on day 4 after extracorporeal cardiopulmonary resuscitation, subcostal view.

Supplementary Video 6 Pre-discharge echocardiography, parasternal long-axis view.

## References

[1] Traub R Wisseman CL Jr. The ecology of chigger-borne rickettsiosis (scrub typhus). J Med Entomol. (1974) 11:237–303. 10.1093/jmedent/11.3.237 4212400

[2] Xu G Walker DH Jupiter D Melby PC Arcari CM. A review of the global epidemiology of scrub typhus. PLoS Negl Trop Dis. (2017) 11:e0006062. 10.1371/journal.pntd.0006062 29099844 PMC5687757

[3] Jiang J Richards AL. Scrub typhus: no longer restricted to the tsutsugamushi triangle. Trop Med Infect Dis. (2018) 3:11. 10.3390/tropicalmed3010011 30274409 PMC6136623

[4] Costa C Ferrari A Binazzi R Beltrame A Tacconi D Moro L. Imported scrub typhus in Europe: report of three cases and a literature review. Travel Med Infect Dis. (2021) 42:102062. 10.1016/j.tmaid.2021.102062 33862243

[5] Kim DM Kim SW Choi SH Yun NR. Clinical and laboratory findings associated with severe scrub typhus. BMC Infect Dis. (2010) 10:108. 10.1186/1471-2334-10-108 20433689 PMC2877676

[6] Peter JV Sudarsan TI Prakash JA Varghese GM. Severe scrub typhus infection: Clinical features, diagnostic challenges and management. World J Crit Care Med. (2015) 4:244–50. 10.5492/wjccm.v4.i3.244 26261776 PMC4524821

[7] Lee N Ip M Wong B Lui G Tsang OT Lai JY. Risk factors associated with life-threatening rickettsial infections. Am J Trop Med Hyg. (2008) 78:973–8. 10.4269/ajtmh.2008.78.973 18541779

[8] Sittiwangkul R Pongprot Y Silviliarat S Oberdorfer P Jittamala P Sirisanthana V. Acute fulminant myocarditis in scrub typhus. Ann Trop Paediatr. (2008) 28:149–54. 10.1179/146532808X302189 18510826

[9] Nakayama K Yamashita A Kurokawa K Morimoto T Ogawa M Fukuhara M. The Whole-genome sequencing of the obligate intracellular bacterium *Orientia tsutsugamushi* revealed massive gene amplification during reductive genome evolution. DNA Res. (2008) 15:185–99. 10.1093/dnares/dsn011 18508905 PMC2575882

[10] Chang WH Kang JS Lee WK Choi MS Lee JH. Serological classification by monoclonal antibodies of *Rickettsia tsutsugamushi* isolated in Korea. J Clin Microbiol. (1990) 28:685–8. 10.1128/jcm.28.4.685-688.1990 2110179 PMC267777

[11] Taylor AJ Paris DH Newton PN. A Systematic Review of Mortality from Untreated Scrub Typhus (*Orientia tsutsugamushi*). PLoS Negl Trop Dis. (2015) 9:e0003971. 10.1371/journal.pntd.0003971 26274584 PMC4537241

[12] Chin JY Kang KW Moon KM Kim J Choi YJ. Predictors of acute myocarditis in complicated scrub typhus: an endemic province in the Republic of Korea. Korean J Intern Med. (2018) 33:323–30. 10.3904/kjim.2016.303 28226202 PMC5840598

[13] Yotsukura M Aoki N Fukuzumi N Ishikawa K. Review of a case of tsutsugamushi disease showing myocarditis and confirmation of Rickettsia by endomyocardial biopsy. Jpn Circ J. (1991) 55:149–53. 10.1253/jcj.55.149 1902270

[14] Ki YJ Kim DM Yoon NR Kim SS Kim CM. A case report of scrub typhus complicated with myocarditis and rhabdomyolysis. BMC Infect Dis. (2018) 18:551. 10.1186/s12879-018-3458-1 30404620 PMC6223005

[15] Jeong MH Ahn YK Gill GC Park JH Cho JG Park JC. Tsutsugamushi myocarditis with congestive heart failure and persistent atrial standstill. Jpn Circ J. (1996) 60:382–8. 10.1253/jcj.60.382 8844306

[16] Jung YH Lee L Lee JH Kim S-H Lee BK. Acute fulminant myocarditis recovered from Electro-Mechanical Dissociation in Scrub Typhus. Ewhan Med J. (2016) 39:1–5. 10.12771/emj.2016.39.1.1

[17] Li S Xu S Li C Ran X Cui G He M. A life support-based comprehensive treatment regimen dramatically lowers the in-hospital mortality of patients with fulminant myocarditis: a multiple center study. Sci China Life Sci. (2019) 62:369–80. 10.1007/s11427-018-9501-9 30850929

[18] Schubert S Opgen-Rhein B Boehne M Weigelt A Wagner R Muller G. Severe heart failure and the need for mechanical circulatory support and heart transplantation in pediatric patients with myocarditis: results from the prospective multicenter registry “MYKKE”. Pediatr Transplant. (2019) 23:e13548. 10.1111/petr.13548 31297930

[19] Korea Centers for Disease Control Prevention. (2018). https://www.kdca.go.kr/gallery.es?mid=a20509000000andbid=0007andact=viewandlist_no=140972 (accessed November 8, 2021).

