# Supplementary figures and images for: Case Report: Fulminant Myocarditis Successfully Treated With Extracorporeal Membrane Oxygenation in Ikeda Strain Orientia tsutsugamushi Infection
Source: Front Cardiovasc Med. 2021 Dec 22;8:795249. doi: 10.3389/fcvm.2021.795249 (PMC8727758; doi:10.3389/fcvm.2021.795249)

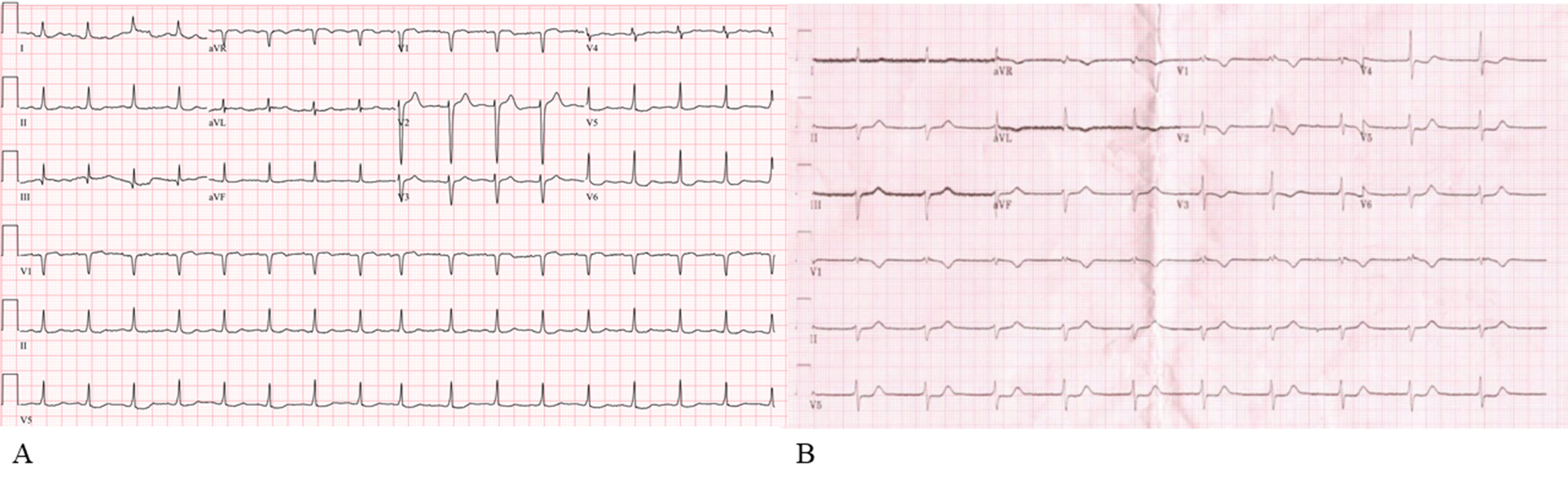

Supplement: Supplementary Figure 1 — (A) EKG at presentation showed slight ST-segment depression on the V5-V6. (B) EKG on hospital day 3 showed newly developed T wave inversion on V1-V3. [file Image_1.TIF]
