# Supplementary material for: Case Report: Fulminant Myocarditis Successfully Treated With Extracorporeal Membrane Oxygenation in Ikeda Strain Orientia tsutsugamushi Infection
Source: Front Cardiovasc Med. 2021 Dec 22;8:795249. doi: 10.3389/fcvm.2021.795249 (PMC8727758; doi:10.3389/fcvm.2021.795249)
Supplement: Supplementary Table 1 — Laboratory findings at initial presentation. [file Table_1.DOCX]

| Supplementary table 1. Laboratory findings at presentation on hospital day 1 | | | | | |
| --- | --- | --- | --- | --- | --- |
|  | | | Value | Unit | Reference value |
| Complete Blood Count | | | | | |
| White blood cell | | | 9,800 | /mL | 4800-10800 |
|  | | Neutrophil (%) | 93.1 | % | 50-75 |
|  | | Monocyte (%) | 2.3 | % | 2-9 |
|  | | Eosinophil (%) | 0 | % | 0-5 |
|  | | Basophil (%) | 0.5 | % | 0-2 |
| Hemoglobin | | | 11.0 | g/dL | 12-18 |
| Platelet | | | 50000 | /mL | 130000-450000 |
| Chemistry | | | | | |
|  | Total protein | | 5.4 | g/dL | 6-8.3 |
|  | Albumin | | 3.0 | g/dL | 3.5-5.2 |
|  | AST | | 188 | U/L | 10-37 |
|  | ALT | | 70 | U/L | 10-37 |
|  | Total bilirubin | | 0.97 | mg/dL | 0.22-1.3 |
|  | Direct bilirubin | | 0.47 | Mg/dL | 0.05-0.3 |
|  | Alkaline phosphatase | | 248 | U/L | 35-129 |
|  | Gamma-GTP | | 134 | U/L | 5-61 |
|  | BUN | | 50.6 | Mg/dL | 8-23 |
|  | Creatinine | | 2.84 | Mg/dL |  |
|  | Sodium | | 138 | mEq/L | 136-146 |
|  | Potassium | | 4.7 | mEq/L | 3.5-5.1 |
|  | Chloride | | 107 | mEq/L | 98-110 |
|  | Creatine kinase | | 842 | IU/L | 0-170 |
|  | C-Reactive protein | | 28.59 | Mg/dL | 0-0.3 |
|  | Procalcitonin | | 30.0 | Ng/mL | 0-0.5 |
|  | CK-MB | | 38.62 | Ng/mL | 0-5 |
|  | High sensitivity Troponin-T | | 0.231 | Ng/mL | 0-0.014 |
|  | aPTT | | 36.3 | Sec | 22.5-34.5 |
|  | Prothrombin time (INR) | | 1.16 | INR |  |
|  | Fibrinogen assay | | 484.6 | Mg/dL | 180-400 |
|  | FDP | | 90.5 | Ug/ml | 0-5 |
|  | D-dimer | | 23.78 | Mg/L FEU | 0-0.55 |
| ALT= Alanine aminotransferase ; aPTT= activated partial thromboplastin time; AST= Aspartate aminotransferase; BUN = Blood urea nitrogen; FDP= Fibrinogen degradation product; GTP=Glutamyl Transferase | | | | | |
